# Comparison of Midterm Outcomes Associated With Aspirin and Ticagrelor vs Aspirin Monotherapy After Coronary Artery Bypass Grafting for Acute Coronary Syndrome

**DOI:** 10.1001/jamanetworkopen.2021.22597

**Published:** 2021-08-26

**Authors:** Erik Björklund, Carl Johan Malm, Susanne J. Nielsen, Emma C. Hansson, Hans Tygesen, Birgitta S. Romlin, Andreas Martinsson, Elmir Omerovic, Aldina Pivodic, Anders Jeppsson

**Affiliations:** 1 Department of Molecular and Clinical Medicine, Institute of Medicine, Sahlgrenska Academy, Gothenburg University, Sweden; 2 Department of Medicine, South Älvsborg Hospital, Borås, Sweden; 3 Department of Cardiothoracic Surgery, Sahlgrenska University Hospital, Gothenburg, Sweden; 4 Department of Anesthesiology and Intensive Care, Sahlgrenska University Hospital, Gothenburg, Sweden; 5 Department of Cardiology, Sahlgrenska University Hospital, Gothenburg, Sweden; 6 Statistiska Konsultgruppen, Gothenburg, Sweden; 7 Department of Ophthalmology, Institute of Neuroscience and Physiology, Sahlgrenska Academy, University of Gothenburg, Gothenburg, Sweden

## Abstract

**Importance:**

Guidelines recommend dual antiplatelet therapy after coronary artery bypass grafting (CABG) for patients with acute coronary syndrome (ACS). However, the evidence for these recommendations is weak.

**Objective:**

To compare midterm outcomes after CABG in patients with ACS treated postoperatively with acetylsalicylic acid (ASA) and ticagrelor or with ASA monotherapy.

**Design, Setting, and Participants:**

This cohort study used merged data from several national registries of Swedish patients who were diagnosed with ACS and subsequently underwent CABG. All included patients underwent isolated CABG in Sweden between 2012 and 2017 with an ACS diagnosis less than 6 weeks before the procedure, survived 14 days after discharge from hospital, and were treated postoperatively with ASA plus ticagrelor or ASA monotherapy. A multivariable Cox regression model was used for the main analysis, and propensity score-matched models were performed as sensitivity analysis. Data were analyzed between May and September 2020.

**Exposures:**

Postoperative antiplatelet treatment, defined as filled prescriptions, with either ASA and ticagrelor or ASA only.

**Main Outcomes and Measures:**

Major adverse cardiovascular events (MACE), defined as all-cause mortality, myocardial infarction, and stroke, and major bleeding, at 12 months and at the end of follow-up.

**Results:**

A total of 6558 patients (5281 [80.5%] men; mean [SD] age at surgery, 67.6 [9.3] years) were included; 1813 (27.6%) were treated with ASA plus ticagrelor and 4745 (72.4%) were treated with ASA monotherapy. Crude MACE rate was 3.0 per 100 person years (95% CI, 2.5-3.6 per 100 person years) in the ASA plus ticagrelor group and 3.8 per 100 person years (95% CI, 3.5-4.1 per 100 person years) in the ASA group. After adjustment, there was no significant difference in MACE risk between ASA plus ticagrelor vs ASA only, neither during the first 12 months (adjusted hazard ratio [aHR], 0.84; 95% CI, 0.58-1.21; *P* = .34) or during total follow-up (aHR, 0.89; 95% CI, 0.71-1.11; *P* = .29). The use of ASA plus ticagrelor was associated with a significantly increased risk for major bleeding during the first 12 months (aHR, 1.90; 95% CI, 1.16-3.13; *P* = .011). Sensitivity analyses confirmed the results.

**Conclusions and Relevance:**

In patients with ACS who survived 2 weeks after CABG, no significant difference in the risk of death or ischemic events could be demonstrated between ASA plus ticagrelor and patients treated with ASA only, while the risk for major bleeding was higher in patients treated with ASA plus ticagrelor. Sufficiently powered prospective randomized trials comparing different antiplatelet therapy strategies after CABG are warranted.

## Key Points

**Question:**

Is dual antiplatelet therapy with acetylsalicylic acid (ASA) and ticagrelor associated with better clinical outcomes for patients with acute coronary syndrome treated with coronary artery bypass grafting compared with ASA monotherapy?

**Findings:**

In this nationwide cohort study including 6558 patients, there was no significant risk reduction in major adverse cardiovascular events (a composite of all-cause mortality, myocardial infarction, and stroke) associated with ASA plus ticagrelor treatment compared with ASA monotherapy, although the dual therapy was associated with increased risk for major bleeding.

**Meaning:**

These results suggest that further research, preferably randomized clinical trials, are warranted to define optimal antiplatelet therapy in this group.

## Teaser

This cohort study compares clinical outcomes among patients with acute coronary syndrome treated with aspirin and ticagrelor vs aspirin alone after artery bypass grafting.

## Introduction

Dual antiplatelet therapy (DAPT) with acetylsalicylic acid (ASA) and a P2Y_12_ inhibitor reduces ischemic events and death in patients with acute coronary syndrome (ACS) and is recommended in the current guidelines for all ACS patients without contraindications, including those undergoing coronary artery bypass grafting (CABG).^1,2^ The recommended duration of DAPT for patients with ACS undergoing CABG is 12 months, and the use of a more potent P2Y_12_ inhibitor (eg, ticagrelor or prasugrel) is recommended over clopidogrel.^1,2^

The guidelines for DAPT for patients with ACS undergoing CABG recommended by both the American College of Cardiology/American Heart Association and European Society of Cardiology^1,2^ are based on limited evidence (level C) mainly derived from non-CABG populations, subgroup analyses of ACS trials,^3,4^ and meta-analyses of small randomized trials with surrogate end points.^5,6,7^ No randomized trials with sufficient statistical power or large observational studies with clinical outcome data supporting the current DAPT guidelines for patients undergoing CABG have been published, and the adherence to DAPT recommendations has consistently been reported to be low.^8,9,10,11^

In this nationwide cohort study of Swedish adults, we investigated whether the adjusted risk of ischemic events and major bleeding after CABG differed between patients with ACS treated with ASA and ticagrelor compared with ASA alone. A secondary aim was to describe the use of DAPT over time in patients with ACS undergoing CABG.

## Methods

### Study Population and Study Design

The study population was identified in the Swedish Cardiac Surgery Registry, which is part of the SWEDEHEART registry.^12,13^ All patients older than 18 years who underwent isolated first-time CABG from January 1, 2006, to December 31, 2017, with an ACS diagnosis within 6 weeks before CABG were considered for inclusion (a total of 10 181 patients). Start of follow-up was 15 days after hospital discharge and end of follow-up was at death, emigration, or after December 31, 2017. The following patients were excluded: patients not surviving until start of follow-up (ie, died during hospital stay or within 14 days after discharge [122 patients]), patients with less than 14 days of follow-up (ie, discharged after December 16, 2017 [79 patients]), patients receiving oral anticoagulants or other P2Y_12_ inhibitors than ticagrelor at the start of follow-up (1775 patients), patients treated with ticagrelor or anticoagulants within 6 months before surgery (972 patients), and patients not taking ASA (675 patients). After exclusion of patients according to eFigure 1 in the Supplement, 6558 patients were included in the analysis. Co-primary end points were time to first major adverse cardiovascular event (MACE; defined as all-cause mortality, myocardial infarction, and stroke) and major bleeding within the first 12 months and at the end of follow-up. The individual components of the primary end point and the composite end point net adverse clinical event (NACE; defined as all-cause mortality, myocardial infarction, stroke, or major bleeding) were analyzed as secondary end points.

### Data Sources and Definitions

Individual patient data from 5 nationwide mandatory registries were merged based on personal identification numbers (given to all Swedish residents at birth or shortly after immigration), which was performed at the National Board of Health and Welfare and Statistics Sweden as previously described.^14^ Data on the CABG procedure, including preoperative status and postoperative complications, were retrieved from the Swedish Cardiac Surgery Registry, which contains information on cardiac surgery procedures in Sweden since 1992 with a coverage of 98% to 99%.^13^ The Chronic Kidney Disease Epidemiology Collaboration formula was used to calculate estimated glomerular filtration rate (eGFR) based on preoperative creatinine levels.^15^ Comorbidities not already registered in the Swedish Cardiac Surgery Registry were collected from the National Patient Register, which has full coverage of diagnoses according to the *International Classification of Diseases, Ninth Revision (ICD-9)* and *ICD-10* from all hospital admissions in Sweden with a validity of 85% to 95%.^16^ All comorbidities registered until start of follow-up were registered as baseline data. Clinical end points of nonfatal myocardial infarction, stroke, and major bleeding were retrieved from the National Patient Register and defined as hospitalization with a main diagnosis of myocardial infarction, stroke, or bleeding. Stroke included both ischemic and hemorrhagic events, and the bleeding end point consisted of a wide spectrum of bleeding diagnosis including pericardial bleeding (see eTable 1 in the Supplement for *ICD* codes used). Mortality data were gathered from the Cause of Death Register.

The Swedish Prescribed Drug Register, which has information on all prescriptions dispensed from Swedish pharmacies from July 2005 according to Anatomical Therapeutic Chemicals classification, was used to obtain data on platelet inhibitors and other secondary prevention medications (see eTable 2 in the Supplement for codes used). Assignment to the ASA group was based on a dispensed prescription of ASA within 6 months before surgery and/or within 14 days after hospital discharge. Assignment to the ASA plus ticagrelor group was based on a dispensed prescription of ASA as described above and a dispensed prescription of ticagrelor from the date of surgery to 14 days after hospital discharge. Exposure status for statins, β-blockers, and renin-angiotensin-aldosterone system (RAAS) inhibitors were updated every third month as described previously.^14^ The Swedish Population Register was used for basic demographic information including potential date of emigration.

This study has been composed according to the Strengthening of Observational Studies in Epidemiology (STROBE) reporting guideline. The study was performed in accordance with the Declaration of Helsinki^17^ and was approved by the Regional Research Ethical Committee in Gothenburg, which waived the need for individual patient consent. All cardiac surgery patients in Sweden are notified that patient data are registered in official quality registries and databases and can be used for research after approval from the research ethics committee.

### Statistical Analysis

Continuous variables are presented as means with standard deviation (SD), medians with range, or medians with interquartile ranges (IQRs) where appropriate. Categorical variables are presented as frequency with percentage. For comparison between 2 groups, a Fisher exact test was used for dichotomous variables, Mantel-Haenszel χ^2^ trend test for ordered categorical variables, χ^2^ test for unordered categorical variables, and Mann-Whitney U test for continuous variables. Use of platelet inhibitors during the first year was calculated by dividing the number of dispensed daily doses with days of follow-up.

Crude event rates per 100 person-years were calculated as the number of events divided by the number of years of follow-up with 95% CIs estimated using exact Poisson limits. Cumulative incidence for the end points were estimated using Kaplan-Meier technique. For myocardial infarction, stroke, and major bleeding, death was handled as a competing risk in the estimation of cumulative incidence. Cox proportional hazards models were used as the main analyses to evaluate the association between use of ticagrelor and the end points described above. A simple model was adjusted for age and sex, and a more complex model was additionally adjusted for year of surgery; left ventricular ejection fraction and eGFR categories; time-updated use of statins, β-blockers, and RAAS inhibitors; and other baseline variables that were associated with any of the studied outcomes based on separately performed stepwise (forward) regression models applying *P* < .10. This approach resulted in adjustments for the use of left internal mammary artery in grafting, smoking, ACS type, body mass index category, previous bleeding, kidney failure, heart failure, hypertension, previous percutaneous coronary intervention, transient ischemic attack, diabetes, atrial fibrillation, chronic obstructive pulmonary disease, peripheral vascular disease, history of cancer, chronic respiratory disease, hyperlipidaemia, ischemic stroke, asthma, and history of stable angina.

For sensitivity analysis, a propensity score model was developed with individual matching based on the same baseline variables as included in the main analysis. Using a caliper width of 0.005, 1359 patients from the ASA plus ticagrelor group were matched 1:1 with patients from the ASA monotherapy group, which resulted in balanced groups regarding the included variables (eTables 3 and 4 in the Supplement). The time-updated use of secondary prevention medications was then adjusted for in the comparison between the matched groups. Standardized mean difference between the groups was calculated before and after the matching and then described. The assumption of proportional hazard was tested including interaction between log-time and treatment group. The *P* value for interaction was > .05 in all analyses, except for the sensitivity analysis of stroke during the complete follow-up (*P* = .045). Interaction analyses were performed for the following subgroups: age (<75 or ≥75 years), sex, presenting with myocardial infarction or unstable angina, history of bleeding, diabetes, peripheral vascular disease, eGFR (≥60 or <60 mL/min/1.73 m^2^), and left ventricular ejection fraction (>50% or ≤50%).

Missing data were handled as a separate category in the adjustments. All tests were 2-tailed and were interpreted at the *P* < .05 significance level. Statistical analyses were performed using SAS Software version 9.4 (SAS Institute Inc).

## Results

### Study Population

The study cohort of 6558 patients consisted of 5281 (80.5%) men and the mean (SD) age was 67.6 (9.3) years. Overall, 4745 patients (72.4%) were treated with ASA monotherapy and 1813 patients (27.6%) were treated with ASA and ticagrelor at baseline. Further baseline patient characteristics are presented in Table 1. Patients treated with ASA plus ticagrelor were younger, more often men, had fewer comorbidities except from previous percutaneous coronary intervention (PCI) and had higher utilization of statins and RAAS inhibitors at baseline than patients with ASA monotherapy. Median (IQR) follow-up was 2.9 (1.4-4.4) years and total follow-up time was 19 111 patient-years. No patient was lost to follow-up. After propensity score matching the groups were balanced regarding all variables included in the matching procedure (eTables 3 and 4, eFigure 2 in the Supplement).

**Table 1.** Patient Characteristics in the ASA and ASA Plus Ticagrelor Groups at Baseline

Characteristics	No. (%)		*P* value
	ASA (n = 4745)	ASA plus ticagrelor (n = 1813)	
Age at surgery, mean (SD), y	68.1 (9.1)	66.3 (9.4)	<.001
Sex			
Men	3790 (79.9)	1491 (82.2)	.03
Women	955 (20.1)	322 (17.8)	
BMI, mean (SD)	27.8 (10.2)	27.5 (4.1)	.36
Missing	404	41	
eGFR, mean (SD), mL/min/1.73 m^2^	76.0 (19.2)	79.4 (17.5)	<.001
Missing	36	11	
Left ventricular ejection fraction			.10
Normal (>50%)	3278 (69.5)	1210 (66.9)	
31%-50%	1210 (25.7)	511 (28.2)	
21%-30%	198 (4.2)	76 (4.2)	
≤20%	30 (0.6)	12 (0.7)	
Missing	29	4	
Type of acute coronary syndrome within 6 wk before surgery			<.001
STEMI	381 (8.0)	230 (12.7)	
NSTEMI	2190 (46.2)	1151 (63.5)	
Unstable angina	2174 (45.8)	432 (23.8)	
Smoking			.82
Never	1401 (30.4)	528 (30.3)	
Previous	2412 (52.4)	908 (52.1)	
Current	794 (17.2)	306 (17.6)	
Missing	138	71	
Baseline comorbidities			
History of ischemic stroke	353 (7.4)	90 (5.0)	<.001
History of transient ischemic attack	282 (5.9)	81 (4.5)	.02
Diabetes	1543 (32.5)	552 (30.4)	.11
Hypertension	3365 (70.9)	1276 (70.4)	.69
Heart failure	724 (15.3)	240 (13.2)	.04
Atrial fibrillation	925 (19.5)	320 (17.7)	.09
History of percutaneous coronary intervention	769 (16.2)	353 (19.5)	.002
Peripheral vascular disease	464 (9.8)	123 (6.8)	<.001
Hyperlipidemia	2014 (42.4)	766 (42.3)	.91
History of cancer	732 (15.4)	248 (13.7)	.08
Chronic respiratory disease	511 (10.8)	160 (8.8)	.02
Euroscore II, median (range), %	1.5 (0.5-57.9)	1.6 (0.5-48.6)	.09
Missing	20	3	
Medications at baseline			
Statins	4541 (95.7)	1763 (97.2)	.004
RAAS inhibitors	3734 (78.7)	1492 (82.3)	.001
β-blockers	4447 (93.7)	1699 (93.7)	>.99

Abbreviations: ASA, acetylsalicylic acid; BMI, body mass index (calculated as weight in kilograms divided by height in meters squared); eGFR, estimated glomerular filtration rate; NSTEMI, non–ST-elevation myocardial infarction; RAAS, renin-angiotensin-aldosterone system; STEMI, ST-elevation myocardial infarction.

### DAPT Utilization Over Time

**Figure 1.**
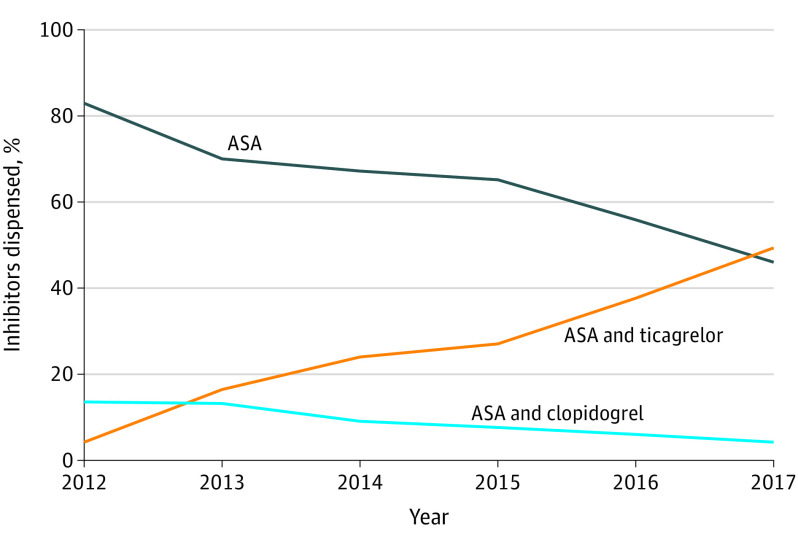
Dispensed Platelet Inhibitors Within 14 Days After Hospital Discharge From CABG, 2012-2017 ASA indicates acetylsalicylic acid; CABG, coronary artery bypass grafting.

The percentage of patients treated with ASA plus ticagrelor increased from 4.2% in 2012 (59 of 1394 patients) to 49.2% in 2017 (516 of 1049 patients), the percentage treated with ASA monotherapy decreased from 82.6% (1151 of 1394 patients) to 46.5% (488 of 1049 patients) and the corresponding figures for ASA plus clopidogrel decreased from 13.2% (184 of 1394 patients) to 4.3% (45 of 1049 patients) (*P* < .001) (Figure 1). Patients receiving clopidogrel were excluded from the outcome analyses. During the first year of follow-up, the dispensed daily doses of ASA and ticagrelor covered 93.4% and 82.2% respectively of the time in the ASA plus ticagrelor group. In the ASA group, dispensed daily doses of ASA covered 89.4% of the time.

### Event Rates

During follow-up, 670 patients (10.2%) suffered a MACE, 366 patients (5.6%) died, 207 patients (3.2%) had a myocardial infarction, 160 patients (2.4%) had a stroke, and 197 patients (3.0%) experienced a major bleeding event. A NACE event occurred in 798 patients (12.2%).

**Figure 2.**
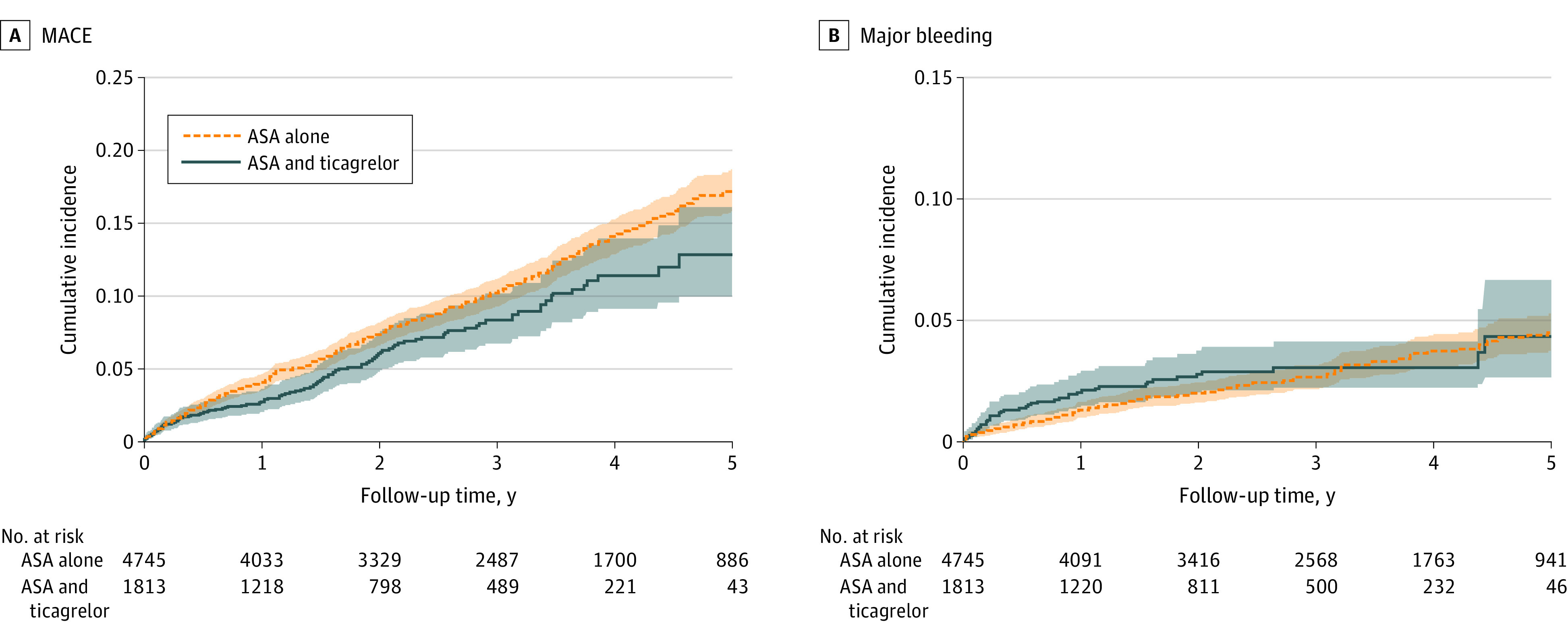
Incidence of MACE and Major Bleeding Events Following CABG The shaded areas represent 95% CIs. ASA indicates acetylsalicylic acid; MACE, major adverse cardiovascular event.

Crude event rates for all end points are presented in Table 2. The crude event rate for MACE was 3.0 (95% CI, 2.5-3.6) per 100 person-years in the ASA plus ticagrelor group and 3.8 (95% CI, 3.5-4.1) in the ASA group (Figure 2). For all-cause mortality the event rate was 1.3 (95% CI, 1.0-1.7) per 100 person-years in the ASA plus ticagrelor group and 2.1 (95% CI, 1.8-2.3) per 100 person years in the ASA group. The crude event rate for major bleeding events during first year of follow-up was 2.2 (95% CI, 1.5-3.1) per 100 person-years in the ASA plus ticagrelor group and 1.3 (95% CI, 1.0-1.7) in the ASA group.

**Table 2.** Event Rates and HRs for Primary and Secondary End Points in Patients With Acute Coronary Syndrome Treated With ASA Plus Ticagrelor or ASA Only After CABG

	Crude event rate per 100 patient years (95% CI)		ASA plus ticagrelor vs ASA			
	ASA	ASA Plus ticagrelor	Adjusted Cox proportional hazards model, HR (95% CI)^a^	*P* value	Propensity score individually matched model, HR (95% CI)^b^	*P* value
**MACE**						
12 mo	4.1 (3.5-4.8)	2.9 (2.1-3.9)	0.84 (0.58-1.21)	.34	0.67 (0.44-1.01)	.06
End of follow-up	3.8 (3.5-4.1)	3.0 (2.5-3.6)	0.89 (0.71-1.11)	.29	0.81 (0.62-1.05)	.11
**All-cause mortality**						
12 mo	1.7 (1.3-2.1)	1.0 (0.6-1.6)	0.82 (0.44-1.51)	.52	0.61 (0.29-1.28)	.19
End of follow-up	2.1 (1.8-2.3)	1.3 (1.0-1.7)	0.79 (0.57-1.09)	.15	0.73 (0.49-1.09)	.13
**Myocardial infarction**						
12 mo	1.4 (1.1-1.8)	1.6 (1.0-2.4)	1.10 (0.64-1.87)	.74	0.90 (0.51-1.59)	.72
End of follow-up	1.1 (0.9-1.3)	1.3 (0.9-1.7)	1.09 (0.75-1.57)	.66	1.00 (0.65-1.53)	>.99
**Stroke**						
12 mo	1.3 (1.0-1.6)	0.5 (0.2-1.0)	0.47 (0.20-1.10)	.08	0.43 (0.17-1.13)	.09
End of follow-up	0.9 (0.7-1.1)	0.7 (0.5-1.0)	0.93 (0.59-1.47)	.75	0.89 (0.52-1.53)	.67
**Major bleeding**						
12 mo	1.3 (1.0-1.7)	2.2 (1.5-3.1)	1.90 (1.16-3.13)	.01	1.34 (0.76-2.36)	.31
End of follow-up	1.0 (0.8-1.2)	1.3 (0.9-1.7)	1.32 (0.91-1.91)	.14	1.14 (0.73-1.78)	.57
**NACE**						
12 mo	5.1 (4.5-5.8)	5.0 (3.9-6.3)	1.11 (0.83-1.50)	.47	0.89 (0.63-1.25)	.50
End of follow-up	4.5 (4.1-4.8)	4.2 (3.5-4.9)	1.02 (0.84-1.24)	.87	0.92 (0.73-1.17)	.49

Abbreviations: ASA, acetylsalicylic acid; CABG, coronary artery bypass grafting; HR, hazard ratio; MACE, major adverse cardiovascular event (all-cause death, myocardial infarction, stroke); NACE, net adverse clinical event (all-cause death, myocardial infarction, stroke, major bleeding).

^a^ Adjusted for age, sex, kidney function, left ventricular ejection fraction, comorbidities at baseline and time-updated use of other secondary prevention medications.

^b^ Individually matched patients on propensity score obtained using the same baseline variables included in the main analysis and adjusted for time-updated use of other secondary prevention medications.

### Associations Between Platelet Inhibitor Strategy and Clinical Outcome

**Figure 3.**
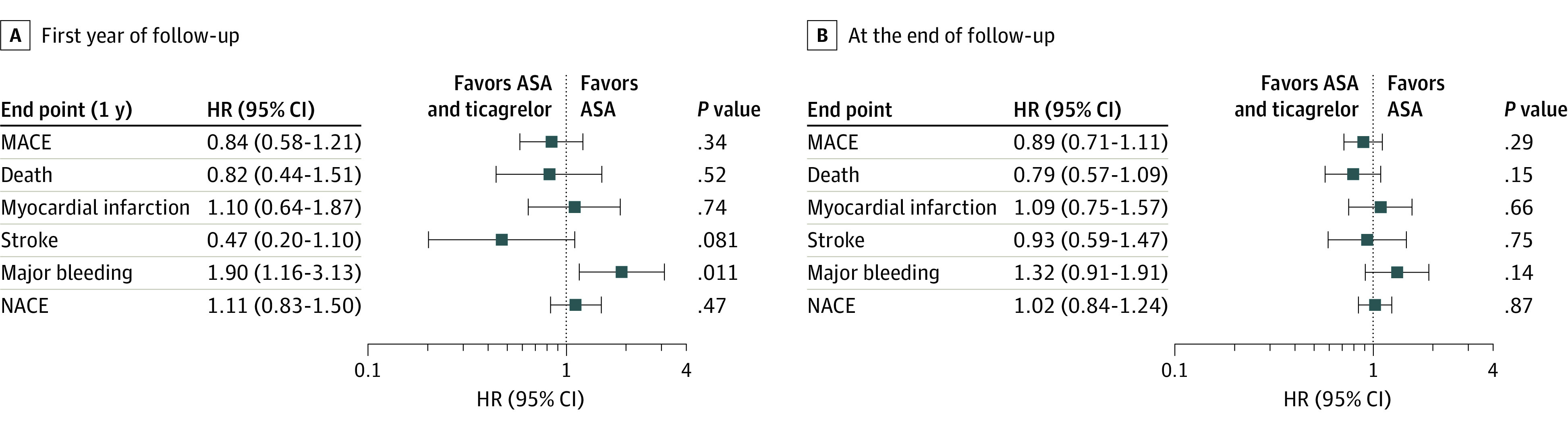
Adjusted Hazard Ratios for MACE, Major Bleeding Events, and NACE During Follow-up MACE indicates major adverse cardiovascular event (all-cause mortality, myocardial infarction, stroke); NACE, net adverse clinical events (all-cause mortality, myocardial infarction, stroke and major bleeding).

In the main analysis adjusted for age, sex, comorbidities at baseline, and time-updated use of other secondary prevention medications there was no difference in MACE, neither during the first year (adjusted hazard ratio [aHR], 0.84; 95% CI, 0.58-1.21; *P* = .34) nor during total follow-up (aHR, 0.89; 95% CI, 0.71-1.11; *P* = .29) (Figure 3). Likewise, there were no significant differences in risk for the individual end points of all-cause death (first year follow-up: aHR, 0.82; 95% CI, 0.44-1.51; *P* = .52; total follow-up: aHR, 0.79; 95% CI, 0.57-1.09; *P* = .15), myocardial infarction (first year: aHR, 1.10; 95% CI, 0.64-1.87; *P* = .74; total: aHR, 1.09; 95% CI, 0.75-1.57; *P* = .66) or stroke (first year: aHR, 0.47; 95% CI, 0.20-1.10; *P* = .08; total: aHR, 0.93; 95% CI, 0.59-1.47; *P* = .75). Treatment with ASA plus ticagrelor was associated with higher risk for major bleeding (aHR, 1.90; 95% CI, 1.16-3.13; *P* = .01) during the first year, but not during the complete follow-up. NACE risk did not differ between ASA plus ticagrelor vs patients treated with ASA only (first year: aHR, 1.11; 95% CI, 0.83-1.50; *P* = .47; total: aHR, 1.02; 95% CI, 0.84 - 1.24; *P* = .87) (Table 2). The results of the age- and sex-adjusted analyses are presented in eTable 5 in the Supplement.

There was no significant interaction for treatment effect on MACE or major bleeding in any of the investigated subgroups (eFigures 4 and 5 in the Supplement). The results of the sensitivity analysis with individually matched patients based on propensity score estimates were in line with the main analyses (Table 2).

## Discussion

There were 3 main findings in this study: (1) the use of DAPT after CABG in Swedish patients with ACS remains limited, (2) there was no significant difference in the risk of MACE between treatment with ASA plus ticagrelor vs ASA only for patients with ACS who underwent CABG, and (3) the use of ASA plus ticagrelor was associated with a higher risk for major bleeding during the first year of follow-up.

### Adherence to Current Guidelines

Current American College of Cardiology/American Heart Association and European Society of Cardiology guidelines recommend 12 months of DAPT to CABG patients with ACS (Class 1 recommendation, level of evidence C).^1,2^ In this study, only 34% of the patients were receiving DAPT (with ASA plus ticagrelor or ASA plus clopidogrel). One may speculate that a lack of strong evidence, fear of bleeding complications, and a strong tradition of prescribing ASA monotherapy to all patients undergoing CABG might contribute to the low adherence. The limited adherence in the present study is in line with other reports from registries and studies.^8,9,11^ Importantly, the adherence increased substantially toward the end of the study period with 53% receiving DAPT with ASA plus ticagrelor or ASA plus clopidogrel during 2017.

### Current Evidence for Postoperative Antiplatelet Therapy

Ticagrelor has been compared with ASA after CABG in a few studies. The randomized TiCAB trial,^18^ which compared ticagrelor and ASA monotherapies and included CABG patients with and without ACS, did not show any difference in MACE at 1 year. The randomized DACAB study^19^ investigated graft patency after CABG comparing DAPT with ASA plus ticagrelor, ticagrelor monotherapy, and ASA monotherapy in patients with stable angina pectoris. The best graft patency was achieved in the DAPT group but there was no significant differences in MACE rate at 1 year.

In contrast to the present study, a Danish registry study^20^ reported that DAPT with ASA plus clopidogrel compared with ASA only was associated with a reduced the risk for death and new myocardial infarction after CABG in patients with myocardial infarction. The results of the present study suggest that ASA monotherapy may not be inferior to DAPT after CABG in patients with ACS, although some caution is warranted. The point estimates indicate an approximately 15% reduced MACE risk in the ASA plus ticagrelor group at 12 months, although this result did not reach statistical significance. It is possible that this risk reduction could reach statistical significance in a larger study population. However, these potential beneficial effects come at the price of a significantly higher bleeding risk. Both bleeding events as such and cessation of DAPT due to bleeding have been associated with increased mortality risk in post-PCI patients.^21,22,23,24,25^ The increased bleeding risk in the ASA plus ticagrelor group during the first 12 postoperative months was expected given the higher intensity of platelet inhibition. A possible mechanism for a less crucial role of DAPT in patients undergoing CABG, compared with ACS patients treated medically or with PCI, might be because of the surgical collateralization protecting against new myocardial infarctions, as recently proposed.^26^

### Methodological Aspects

In line with previous reports demonstrating that severe bleedings factor prominently in prognosis^22,24,25^ we chose only to include major bleeding events, which in our cohort were defined as hospitalization with a main diagnosis of bleeding. Furthermore, we excluded patients treated with ASA plus prasugrel and ASA plus clopidogrel from the outcome analyses based on 2 reasons. First, only 23 patients were treated with ASA plus prasugrel during the study period. Second, comparing patient characteristics and comorbidity in patients treated with ASA plus clopidogrel or ASA plus ticagrelor, it became evident that patients receiving ASA plus clopidogrel were a highly selected patient group with high ischemic risk but were not eligible for ticagrelor treatment because of a perceived higher bleeding risk and frailty. We therefore chose to exclude this group from the outcome analyses because of the substantial risk for bias without possibility for adjustment. Baseline characteristics for the ASA only, the ASA plus clopidogrel, and the ASA plus ticagrelor groups are presented in eTable 6 in the Supplement, and adjusted outcomes for ASA plus clopidogrel vs ASA only in eTable 7 in the Supplement.

### Limitations

This study had several limitations. Despite collecting all eligible patients during a 6-year period in a study that to our knowledge is by far the largest to have compared ASA plus ticagrelor with ASA monotherapy in patients with ACS following CABG, there remains a risk for statistical type II errors resulting from low event rates and a limited study population. Like all observational studies, there is the risk for selection bias and residual confounding. The adherence to DAPT recommendations in Sweden may not be representative for other countries, and we had no information on the reason for patients with ACS receiving or not receiving DAPT. Our database is lacking detailed information about the prevalence of various CABG strategies and graft configurations except for the use of left internal mammary artery and the total number of anastomoses. The 14-day grace period between hospital discharge and start of follow-up infers a small risk for bias, but is necessary given the temporal resolution of the database. The median follow-up time was restricted to 2.9 years.

Strengths of this study include the large study population, its real-world setting, the nationwide coverage, and the completeness of data from validated registries and databases. Despite the limitations above, the study is to our knowledge the first large-population real-world comparison between DAPT with ASA plus ticagrelor and ASA monotherapy in patients with ACS following CABG. In addition, the study provides essential information for designing prospective randomized trials comparing different antiplatelet strategies after CABG.

## Conclusions

This large population-based study demonstrates that the adherence to guideline recommendations for DAPT after CABG in patients with ACS is limited. The event rate after CABG in patients with ACS who survive 2 weeks after hospital discharge is low independent of platelet inhibition strategy. No significant difference in the adjusted risk of ischemic events between treatment with ASA plus ticagrelor and ASA only could be demonstrated, while the bleeding risk during the first postoperative year was increased with ASA plus ticagrelor. Sufficiently powered prospective randomized trials with clinically important end points that compare different antiplatelet strategies after CABG are warranted.

## Notes

Supplement. **eTable 1.***ICD* Codes Used for Defining Comorbidities **eTable 2.** ATC Codes Used for Defining Pharmacotherapies **eTable 3.** Patient Characteristics at Baseline After Propensity Score Matching Using 0.005 Caliper Width **eTable 4.** Standardized Mean Differences in ASA and ASA+Ticagrelor Patients Before and After Propensity Score Matching **eTable 5.** Association Between Platelet Inhibition Treatment and Outcome in an Age- and Sex-Adjusted Model **eTable 6.** Baseline Characteristics for Patients Treated With ASA Alone, ASA+Ticagrelor, and ASA+Clopidogrel **eTable 7.** Adjusted Cox Proportional Hazard Models Comparing ASA+Clopidogrel vs ASA Only at One Year and at End of Follow-up **eFigure 1.** Flowchart Over Included and Excluded Patients **eFigure 2.** Graph Showing Standardized Mean Difference Before and After Propensity Score Matching **eFigure 3.** Cumulative Incidence of Secondary End Points by Treatment Group **eFigure 4.** Forest Plot Describing Multivariable Adjusted Interaction Analyses for Major Adverse Cardiovascular Events (MACE) During Total Follow-up in Predefined Subgroups **eFigure 5.** Forest Plot Describing Multivariable Adjusted Interaction Analyses for Major Bleeding During Total Follow-up in Predefined Subgroups
