# Supplementary material for: Comparison of Midterm Outcomes Associated With Aspirin and Ticagrelor vs Aspirin Monotherapy After Coronary Artery Bypass Grafting for Acute Coronary Syndrome
Source: JAMA Netw Open. 2021 Aug 26;4(8):e2122597. doi: 10.1001/jamanetworkopen.2021.22597 (PMC8391102; doi:10.1001/jamanetworkopen.2021.22597)
Supplement: Supplement. — eTable 1.ICD Codes Used for Defining Comorbidities eTable 2. ATC Codes Used for Defining Pharmacotherapies eTable 3. Patient Characteristics at Baseline After Propensity Score Matching Using 0.005 Caliper Width eTable 4. Standardized Mean Differences in ASA and ASA+Ticagrelor Patients Before and After Propensity Score Matching eTable 5. Association Between Platelet Inhibition Treatment and Outcome in an Age- and Sex-Adjusted Model eTable 6. Baseline Characteristics for Patients Treated With ASA Alone, ASA+Ticagrelor, and ASA+Clopidogrel eTable 7. Adjusted Cox Proportional Hazard Models Comparing ASA+Clopidogrel vs ASA Only at One Year and at End of Follow-up eFigure 1. Flowchart Over Included and Excluded Patients eFigure 2. Graph Showing Standardized Mean Difference Before and After Propensity Score Matching eFigure 3. Cumulative Incidence of Secondary End Points by Treatment Group eFigure 4. Forest Plot Describing Multivariable Adjusted Interaction Analyses for Major Adverse Cardiovascular Events (MACE) During Total Follow-up in Predefined Subgroups eFigure 5. Forest Plot Describing Multivariable Adjusted Interaction Analyses for Major Bleeding During Total Follow-up in Predefined Subgroups [file jamanetwopen-e2122597-s001.pdf]

Supplemental Online Content

Björklund E, Malm CJ, Nielsen SJ, et al. Comparison of midterm outcomes associated with aspirin and ticagrelor vs aspirin monotherapy after coronary artery bypass grafting for acute coronary syndrome. *JAMA Netw Open.* 2021;4(8):e2122597. doi:10.1001/jamanetworkopen.2021.22597

eTable 1. *ICD* Codes Used for Defining Comorbidities

eTable 2. ATC Codes Used for Defining Pharmacotherapies

eTable 3. Patient Characteristics at Baseline After Propensity Score Matching Using 0.005 Caliper Width

eTable 4. Standardized Mean Differences in ASA and ASA+Ticagrelor Patients Before and After Propensity Score Matching

eTable 5. Association Between Platelet Inhibition Treatment and Outcome in an Age- and Sex-Adjusted Model

eTable 6. Baseline Characteristics for Patients Treated With ASA Alone, ASA+Ticagrelor, and ASA+Clopidogrel

eTable 7. Adjusted Cox Proportional Hazard Models Comparing ASA+Clopidogrel vs ASA Only at One Year and at End of Follow-up

eFigure 1. Flowchart Over Included and Excluded Patients

eFigure 2. Graph Showing Standardized Mean Difference Before and After Propensity Score Matching

eFigure 3. Cumulative Incidence of Secondary End Points by Treatment Group

eFigure 4. Forest Plot Describing Multivariable Adjusted Interaction Analyses for Major Adverse Cardiovascular Events (MACE) During Total Follow-up in Predefined Subgroups

eFigure 5. Forest Plot Describing Multivariable Adjusted Interaction Analyses for Major Bleeding During Total Follow-up in Predefined Subgroups

This supplemental material has been provided by the authors to give readers additional information about their work.

eTable 1. *ICD* Codes Used for Defining Comorbidities

Baseline comorbidities	ICD-9	ICD-10
**Ischaemic stroke**	434, 436	I63, I64
**Transient ischaemic attack**	435, 436	I65, I66
**Diabetes**	250	E10-E14
**Hypertension**	401-405	I10-I15
**Heart failure**	425, 428	I42, I43, I50
**Atrial fibrillation**	427D	I48
**Previous bleeding**	280, 282, 285-287, 362W, 430-432, 530C, 531-533, 578A-B, 599H, 626G, 626W, 626X, 719B, 784H, 784W, 786D	D50, D62, D68-D69, H356, H922, I230, I312, I60-I62, I690-I692,I850, I983, J942, K221, K226, K25-K28, K290, K625, K661, K920, K921, K922, M250, N02, N398, N421, N501A, N939, N950, R31, R040-R042, R048, R049
**Renal failure**	584-586	N17-N19
**Peripheral vascular disease**	440-444	I70-I74, I77
**Hyperlipidemia**		E78
**Stable angina**	413	I200, I201
**History of cancer**	200-208	C
**Chronic respiratory disease**	490-496	J40-J47
**Asthma**		J45, J469
**Chronic obstructive pulmonary disease**		J44
**Events**		
**Myocardial infarction**	410	I210, I211, I212, I213, I214, I219
**NSTEMI**	410B	I214
**STEMI**	410A	I210-I213
**Unstable Angina**	411B	I200-I201
**Stroke**		I61-I64, I69
**Bleeding**		D50, D62, D68-D69, H356, H922, I230, I312, I60-I62, I690-I692,I850, I983, J942, K221, K226, K25-K28, K290, K625, K661, K920, K921, K922, M250, N02, N398, N421, N501A, N939, N950, R31, R040-R042, R048, R049

© 2021 Björklund E et al. *JAMA Network Open.*

eTable 2. ATC Codes Used for Defining Pharmacotherapies

	**ATC-codes**
**Statins**	C10AA, C10BA02, C10BX06
**β-blockers**	C07 (not including C07AA07)
**Renin-angiotensin-aldosterone system inhibitors**	C09
**Acetylsalicylic acid**	B01AC06
**Clopidogrel**	B01AC04
**Prasugrel**	B01AC22
**Ticagrelor**	B01AC24
**Oral anticoagulants**	B01AA03, B01AE07, B01AF01, B01AF02, B01AF03

© 2021 Björklund E et al. *JAMA Network Open.*

eTable 3. Patient Characteristics at Baseline After Propensity Score Matching Using 0.005 Caliper Width

	ASA alone (n=1359)	ASA+Tica (n=1359)	p-value
**Age at surgery (years)**	67.0 (9.5)	66.7 (9.3)	1.00
**Sex**			
**Men**	1089 (80.1%)	1108 (81.5%)	
**Women**	270 (19.9%)	251 (18.5%)	0.38
**BMI (kg/m^2)**	27.7 (7.0)	27.5 (4.2)	1.00
**Missing**	43	40	
**eGFR (mL/min)**	78.0 (18.9)	78.9 (18.0)	1.00
**Missing**	6	8	
**LVEF (%)**			
**Normal (EF >50%)**	910 (67.1%)	903 (66.6%)	
**EF 31%-50%**	375 (27.7%)	379 (28.0%)	
**EF 21%-30%**	61 (4.5%)	64 (4.7%)	
**EF <=20%**	10 (0.7%)	9 (0.7%)	0.82
**Missing**	3	4	
**Type of acute coronary syndrome within 6 weeks before surgery**			
**STEMI**	149 (11.0%)	146 (10.7%)	
**NSTEMI**	791 (58.2%)	798 (58.7%)	
**Unstable angina**	419 (30.8%)	415 (30.5%)	0.96
**Smoking**			
**Never smoked**	409 (31.1%)	398 (30.4%)	
**Previous smoker**	669 (50.9%)	676 (51.6%)	
**Current smoker**	237 (18.0%)	235 (18.0%)	0.81
**Missing**	44	50	
**Baseline comorbidities**			
**Previous myocardial infarction**	185 (13.6%)	152 (11.2%)	0.062
**History of ischemic stroke**	88 (6.5%)	80 (5.9%)	0.58
**Transient ischemic attack**	65 (4.8%)	65 (4.8%)	1.00
**Diabetes**	444 (32.7%)	441 (32.5%)	0.93
**Hypertension**	975 (71.7%)	957 (70.4%)	0.47
**Heart failure**	198 (14.6%)	196 (14.4%)	0.96
**Atrial fibrillation**	256 (18.8%)	257 (18.9%)	1.00
**History of percutaneous coronary intervention**	248 (18.2%)	250 (18.4%)	0.96
**Peripheral vascular disease**	91 (6.7%)	107 (7.9%)	0.27
**Hyperlipidemia**	553 (40.7%)	586 (43.1%)	0.21
**Stable angina**	500 (36.8%)	497 (36.6%)	0.94
**History of cancer**	193 (14.2%)	191 (14.1%)	0.96
**Chronic respiratory disease**	126 (9.3%)	131 (9.6%)	0.79
**Euroscore II (%)**	1.6 (0.5; 57.7)	1.6 (0.5; 48.6)	1.00
**Medications at baseline**			
**Statin use at baseline**	1306 (96.1%)	1324 (97.4%)	0.065

© 2021 Björklund E et al. *JAMA Network Open.*

	ASA alone (n=1359)	ASA+Tica (n=1359)	p-value
**RAAS inhibitors use at baseline**	1086 (79.9%)	1117 (82.2%)	0.14
**Beta-blockers use at baseline**	1272 (93.6%)	1270 (93.5%)	0.94

BMI, body mass index; LVEF, left ventricular ejection fraction; NSTEMI, non-ST elevation myocardial infarction; RAAS, renin-angiotensin-aldosterone system; STEMI, ST elevation myocardial infarction

For categorical variables n (%) is presented.
For continuous variables Mean (SD) / Median (Min; Max)
For comparison between groups Fisher´s Exact test (lowest 1-sided p-value multiplied by 2) was used for dichotomous variables and the Mantel-Haenszel Chi Square test was used for ordered categorical variables and Chi Square test was used for non-ordered categorical variables and the Mann-Whitney U-test was used for continuous variables.

© 2021 Björklund E et al. *JAMA Network Open.*

eTable 4. Standardized Mean Differences in ASA and ASA+Ticagrelor Patients Before and After Propensity Score Matching

Variable	SMD Before PS matching	SMD After PS matching
**Age (years)**	0.1941	0.0301
**Sex**	0.0604	0.0355
**Estimated glomerular filtration rate**	0.1567	0.0004
**Left ventricular ejection fraction**	0.0448	0.0089
**Bleeding**	0.0932	0.0186
**Use of left internal mammary artery**	0.0883	0.0100
**Renal failure**	0.0857	0.0235
**Heart failure**	0.0578	0.0042
**Hypertension**	0.0118	0.0292
**History of percutaneous coronary intervention**	0.0853	0.0038
**History of transient ischemic attack**	0.0665	0.0000
**Smoking**	0.0064	0.0091
**Type of acute coronary syndrome**	0.4355	0.0012
**Diabetes**	0.0446	0.0047
**Atrial fibrillation**	0.0474	0.0019
**Chronic obstructive pulmonary disease**	0.0805	0.0350
**Peripheral vascular disease**	0.1088	0.0453
**History of cancer**	0.0496	0.0042
**Chronic respiratory disease**	0.0654	0.0126
**Hyperlipidemia**	0.0039	0.0492
**History of ischemic stroke**	0.1028	0.0244
**Asthma**	0.0284	0.0103
**Stable angina**	0.2482	0.0046
**BMI**	0.0122	0.0009
**Year for surgery**	0.0122	0.0336
BMI, body mass index; PS, propensity score; SMD, standardized mean difference		

© 2021 Björklund E et al. *JAMA Network Open.*

eTable 5. Association Between Platelet Inhibition Treatment and Outcome in an Age- and Sex-Adjusted Model

ASA+ticagrelor vs ASA					
	n (%) events		Age- and sex-adjusted model		
**Outcome**	**ASA**	**ASA+ Tica**	**Hazard Ratio (95% CI)**	**P-value**	**PH assumption P-value**
**NACE (all-cause mortality/MI/stroke/major bleeding)**	650 (13.7%)	148 (8.2%)	0.98 (0.81 - 1.17)	0.79	0.18 Neg
**NACE (all-cause mortality/MI/stroke/major bleeding) during 1 year**	223 (4.7%)	74 (4.1%)	1.03 (0.79 - 1.34)	0.85	0.20 Neg
**All-cause mortality**	317 (6.7%)	49 (2.7%)	0.74 (0.55 - 1.01)	0.057	0.63 Neg
**All-cause mortality during 1st year**	74 (1.6%)	15 (0.8%)	0.66 (0.38 - 1.15)	0.14	0.27 Neg
**Myocardial infarction**	161 (3.4%)	46 (2.5%)	1.09 (0.79 - 1.53)	0.59	0.56 Neg
**Myocardial infarction during 1st year**	63 (1.3%)	24 (1.3%)	1.09 (0.68 - 1.75)	0.71	0.48 Neg
**Stroke**	134 (2.8%)	26 (1.4%)	0.83 (0.54 - 1.27)	0.38	0.098 Pos
**Stroke during 1st year**	56 (1.2%)	7 (0.4%)	0.39 (0.18 - 0.86)	0.019	0.10 Neg
**MACE (all-cause mortality/MI/stroke)**	561 (11.8%)	109 (6.0%)	0.84 (0.68 - 1.03)	0.10	0.71 Neg
**MACE (all-cause mortality/MI/stroke) during 1st year**	181 (3.8%)	44 (2.4%)	0.74 (0.54 - 1.04)	0.080	0.065 Neg
**Major bleeding**	151 (3.2%)	46 (2.5%)	1.29 (0.92 - 1.81)	0.13	0.098 Neg
**Major bleeding during 1st year**	59 (1.2%)	33 (1.8%)	1.77 (1.15 - 2.71)	0.009	0.93 Neg
ASA, acetylsalicylic acid; MACE, major adverse cardiovascular event; MI, myocardial infarction; NACE, net adverse clinical events; Tica, ticagrelor; PH, proportional hazard					

© 2021 Björklund E et al. *JAMA Network Open.*

eTable 6. Baseline Characteristics for Patients Treated With ASA Alone, ASA+Ticagrelor, and ASA+Clopidogrel

				Test between groups p-value		
	ASA alone (n=4745)	ASA+Ticagrelor (n=1813)	ASA+Clopidogrel (n=663)	ASA alone vs ASA+Tica	ASA alone vs ASA+Clopi	ASA+Tica vs ASA+Clopi
**Age at surgery (years)**	68.1 (9.1)	66.3 (9.4)	67.7 (9.0)	<.0001	0.35	0.0019
**Sex**						
**Men**	3790 (79.9%)	1491 (82.2%)	536 (80.8%)			
**Women**	955 (20.1%)	322 (17.8%)	127 (19.2%)	0.032	0.60	0.46
**BMI (kg/m^2)**	27.8 (10.2)	27.5 (4.1)	27.5 (4.2)	0.36	0.75	0.78
**Missing**	404	41	21			
**eGFR (mL/min)**	76.0 (19.2)	79.4 (17.5)	75.1 (19.1)	<.0001	0.12	<.0001
**Missing**	36	11	2			
**LVEF (%)**						
**Normal (EF >50%)**	3278 (69.5%)	1210 (66.9%)	392 (59.8%)			
**EF 31%-50%**	1210 (25.7%)	511 (28.2%)	225 (34.4%)			
**EF 21%-30%**	198 (4.2%)	76 (4.2%)	33 (5.0%)			
**EF <=20%**	30 (0.6%)	12 (0.7%)	5 (0.8%)	0.10	<.0001	0.0037
**Missing**	29	4	8			
**Type of acute coronary syndrome within 6 weeks before surgery**						
**STEMI**	355 (7.6%)	205 (11.5%)	58 (8.9%)			
**NSTEMI**	2164 (46.2%)	1141 (63.9%)	397 (60.6%)			
**Unstable angina**	2164 (46.2%)	439 (24.6%)	200 (30.5%)	<.0001	<.0001	0.0056
**Smoking**						
**Never smoked**	1401 (30.4%)	528 (30.3%)	207 (32.0%)			
**Previous smoker**	2412 (52.4%)	908 (52.1%)	307 (47.5%)			
**Current smoker**	794 (17.2%)	306 (17.6%)	132 (20.4%)	0.82	0.58	0.72
**Missing**	138	71	17			
**Baseline comorbidities**						
**Ischemic stroke**	353 (7.4%)	90 (5.0%)	68 (10.3%)	0.0003	0.017	<.0001
**Transient ischemic attack**	282 (5.9%)	81 (4.5%)	44 (6.6%)	0.021	0.53	0.042
**Diabetes**	1543 (32.5%)	552 (30.4%)	240 (36.2%)	0.11	0.066	0.0080
**Hypertension**	3365 (70.9%)	1276 (70.4%)	487 (73.5%)	0.69	0.19	0.15
**Heart failure**	724 (15.3%)	240 (13.2%)	120 (18.1%)	0.041	0.070	0.0034
**Atrial fibrillation**	925 (19.5%)	320 (17.7%)	155 (23.4%)	0.094	0.024	0.0019
**History of PCI**	769 (16.2%)	353 (19.5%)	136 (20.5%)	0.0021	0.0075	0.60
**Peripheral vascular disease**	464 (9.8%)	123 (6.8%)	67 (10.1%)	0.0001	0.83	0.0091
**Hyperlipidemia**	2014 (42.4%)	766 (42.3%)	298 (44.9%)	0.91	0.24	0.25
**History of cancer**	732 (15.4%)	248 (13.7%)	96 (14.5%)	0.081	0.57	0.65
**Chronic respiratory disease**	511 (10.8%)	160 (8.8%)	78 (11.8%)	0.021	0.48	0.037
**Euroscore II (%)**	1.5 (0.005; 0.579)	1.6 (0.5; 48.6)	1.8 (0.5; 55.8)	0.085	<.0001	<.0001
**Missing**	20	3	7			
**Medications at baseline**						
**Statin use at baseline**	4541 (95.7%)	1763 (97.2%)	625 (94.3%)	0.0037	0.12	0.0010
**RAAS inhibitors use at baseline**	3734 (78.7%)	1492 (82.3%)	526 (79.3%)	0.0012	0.75	0.11
**Beta-blockers use at baseline**	4447 (93.7%)	1699 (93.7%)	635 (95.8%)	1.00	0.039	0.058

ASA, acetylsalicylic acid; Clopi, clopidogrel; BMI, body mass index; LVEF, left ventricular ejection fraction; NSTEMI, non-ST elevation myocardial infarction; PCI, percutaneous coronary intervention; RAAS, renin-angiotensin-aldosterone system; STEMI, ST elevation myocardial infarction; Tica, ticagrelor

For categorical variables n (%) is presented. For continuous variables Mean (SD) / Median (Min; Max) / n= is presented.
For comparison between groups Fisher´s Exact test (lowest 1-sided p-value multiplied by 2) was used for dichotomous variables and the Mantel-Haenszel Chi Square test was used for ordered categorical variables and Chi Square test was used for non-ordered categorical variables and the Mann-Whitney U-test was used for continuous variables.

© 2021 Björklund E et al. *JAMA Network Open.*

eTable 7. Adjusted Cox Proportional Hazard Models Comparing ASA+Clopidogrel vs ASA Only at One Year and at End of Follow-up

			ASA+Clopi vs ASA				
	n (%) events		Age- and sex-adjusted model			Multivariable-adjusted model	
**Outcome**	**ASA**	**ASA+Clopi**	**Hazard Ratio (95% CI)**	**P-value**	**PH assumption P-value**	**Hazard Ratio (95% CI)**	**P-value**
**NACE (all-cause mortality/MI/stroke/major bleeding) during 1 year**	223 (4.7%)	49 (7.4%)	1.62 (1.19 - 2.21)	0.0021	0.59 Pos	1.38 (1.00 - 1.90)	0.053
**MACE (all-cause mortality/MI/stroke) during 1 year**	181 (3.8%)	39 (5.9%)	1.58 (1.12 - 2.23)	0.0096	0.78 Pos	1.30 (0.90 - 1.86)	0.16
**All-cause mortality during 1 year**	74 (1.6%)	14 (2.1%)	1.40 (0.79 - 2.48)	0.25	0.66 Neg	1.21 (0.67 - 2.21)	0.53
**Myocardial infarction during 1 year**	63 (1.3%)	22 (3.3%)	2.53 (1.56 - 4.11)	0.0002	0.86 Pos	2.01 (1.20 - 3.36)	0.0080
**Stroke during 1 year**	56 (1.2%)	4 (0.6%)	0.52 (0.19 - 1.43)	0.20	0.12 Pos	0.41 (0.15 - 1.16)	0.093
**Major bleeding during 1 year**	59 (1.2%)	12 (1.8%)	1.52 (0.82 - 2.83)	0.19	0.82 Pos	1.46 (0.76 - 2.82)	0.26
**NACE (all-cause mortality/MI/stroke/major bleeding)**	650 (13.7%)	122 (18.4%)	1.33 (1.10 - 1.62)	0.0038	0.32 Neg	1.22 (1.00 - 1.49)	0.052
**MACE (all-cause mortality/MI/stroke)**	561 (11.8%)	109 (16.4%)	1.37 (1.11 - 1.68)	0.0027	0.49 Neg	1.24 (1.00 - 1.54)	0.047
**All-cause mortality**	317 (6.7%)	60 (9.0%)	1.31 (0.99 - 1.72)	0.058	0.65 Neg	1.24 (0.93 - 1.66)	0.15
**Myocardial infarction**	161 (3.4%)	44 (6.6%)	1.92 (1.37 - 2.68)	0.0001	0.38 Neg	1.74 (1.22 - 2.47)	0.0020
**Stroke**	134 (2.8%)	16 (2.4%)	0.82 (0.49 - 1.37)	0.44	0.089 Pos	0.66 (0.38 - 1.12)	0.12
**Major bleeding**	151 (3.2%)	17 (2.6%)	0.79 (0.48 - 1.30)	0.35	0.051 Neg	0.74 (0.44 - 1.23)	0.24
ASA, acetylsalicylic acid; MACE, major adverse cardiovascular event; MI, myocardial infarction; NACE, net adverse clinical events; Tica, ticagrelor; PH, proportional hazard							

© 2021 Björklund E et al. *JAMA Network Open.*

eFigure 1. Flowchart Over Included and Excluded Patients

ACS, acute coronary syndrome; ASA, acetylsalicylic acid; CABG, coronary artery bypass grafting

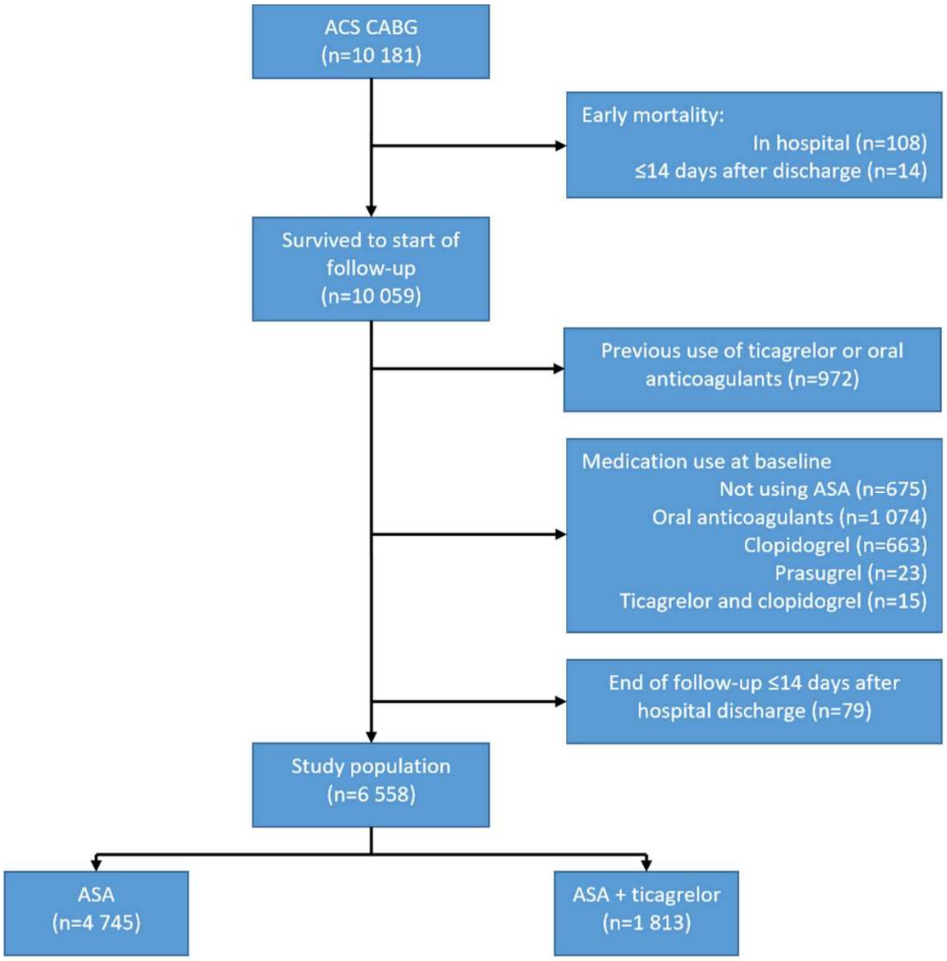

© 2021 Björklund E et al. *JAMA Network Open.*

eFigure 2. Graph Showing Standardized Mean Difference Before and After Propensity Score Matching

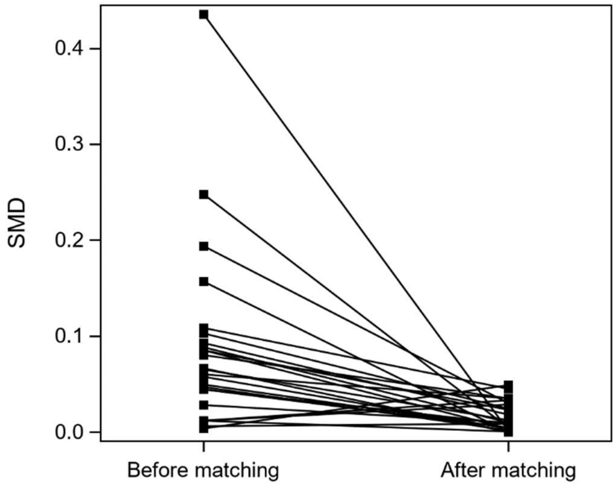

SMD, standardized mean difference.

© 2021 Björklund E et al. *JAMA Network Open.*

eFigure 3. Unadjusted Cumulative Incidence of Secondary End Points by Treatment Group
(A) All-cause mortality, (B) myocardial infarction, (C) stroke, (D) net adverse clinical events (NACE). The shaded areas represent the 95% confidence interval. ASA, acetylsalicylic acid, Tica, ticagrelor

A

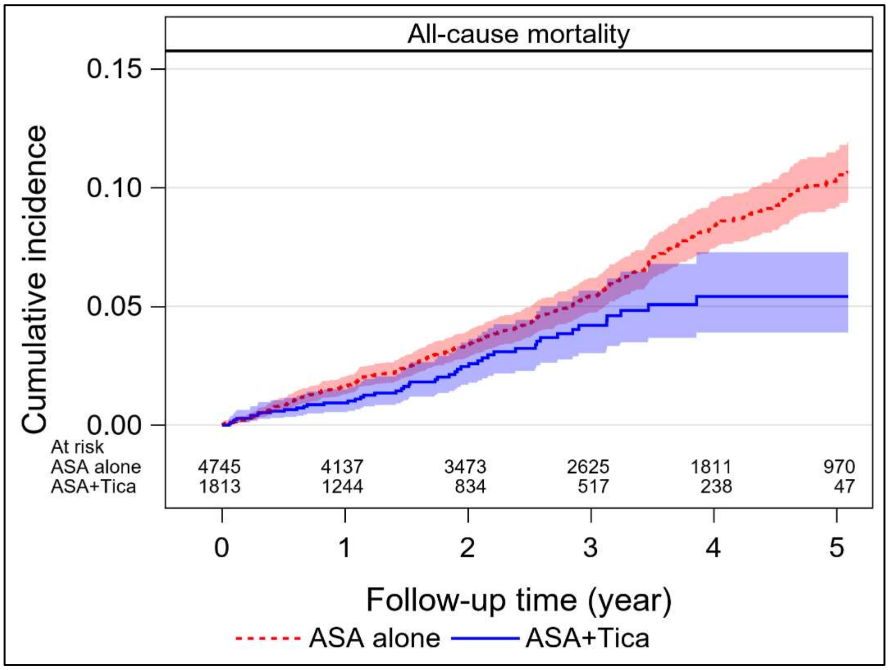

B

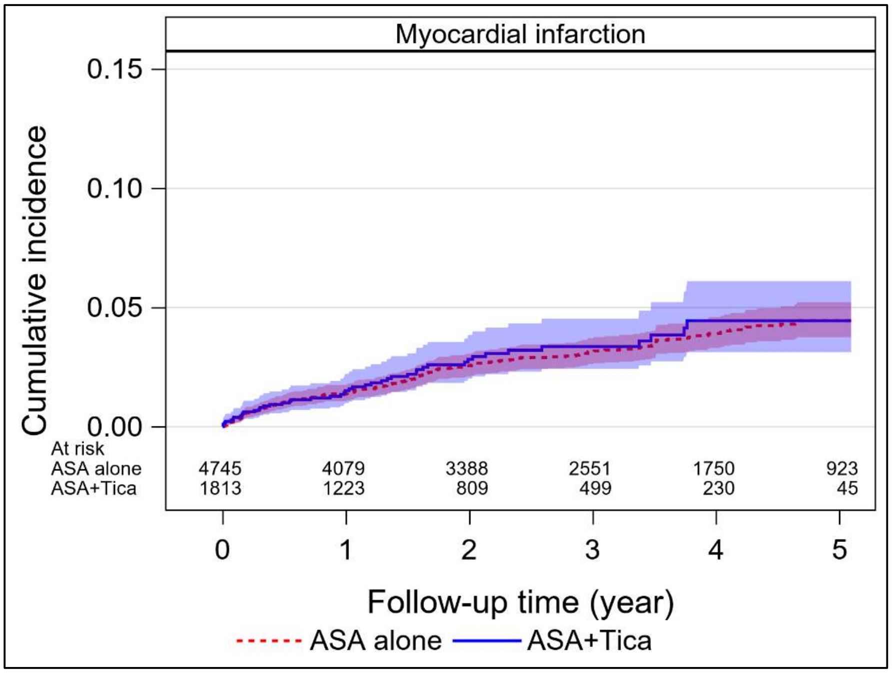

© 2021 Björklund E et al. *JAMA Network Open.*

C

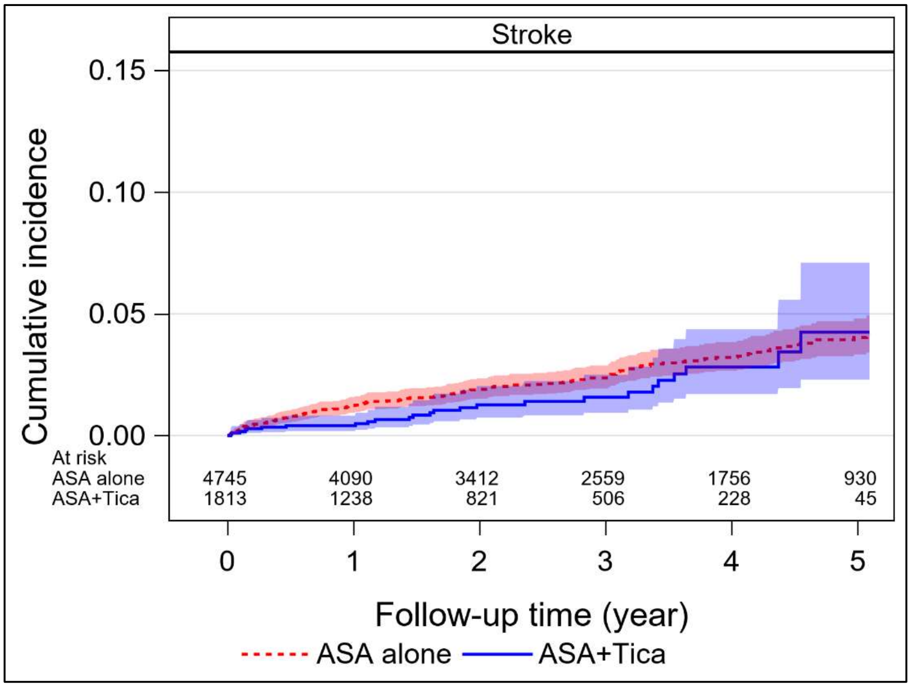

D

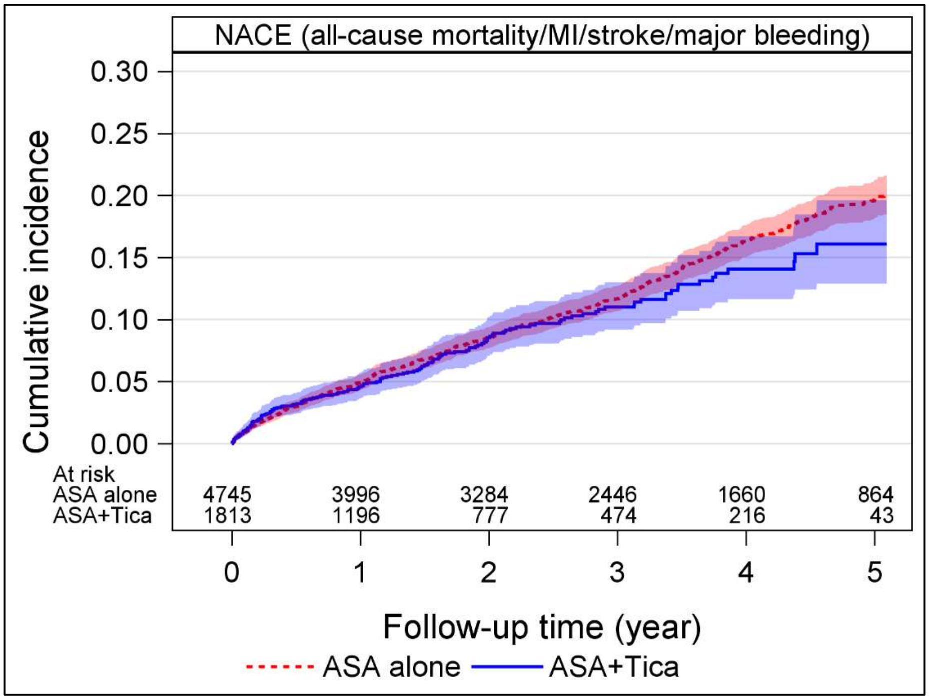

© 2021 Björklund E et al. *JAMA Network Open.*

eFigure 4. Forest Plot Describing Multivariable Adjusted Interaction Analyses for Major Adverse Cardiovascular Events (MACE) During Total Follow-up in Predefined Subgroups

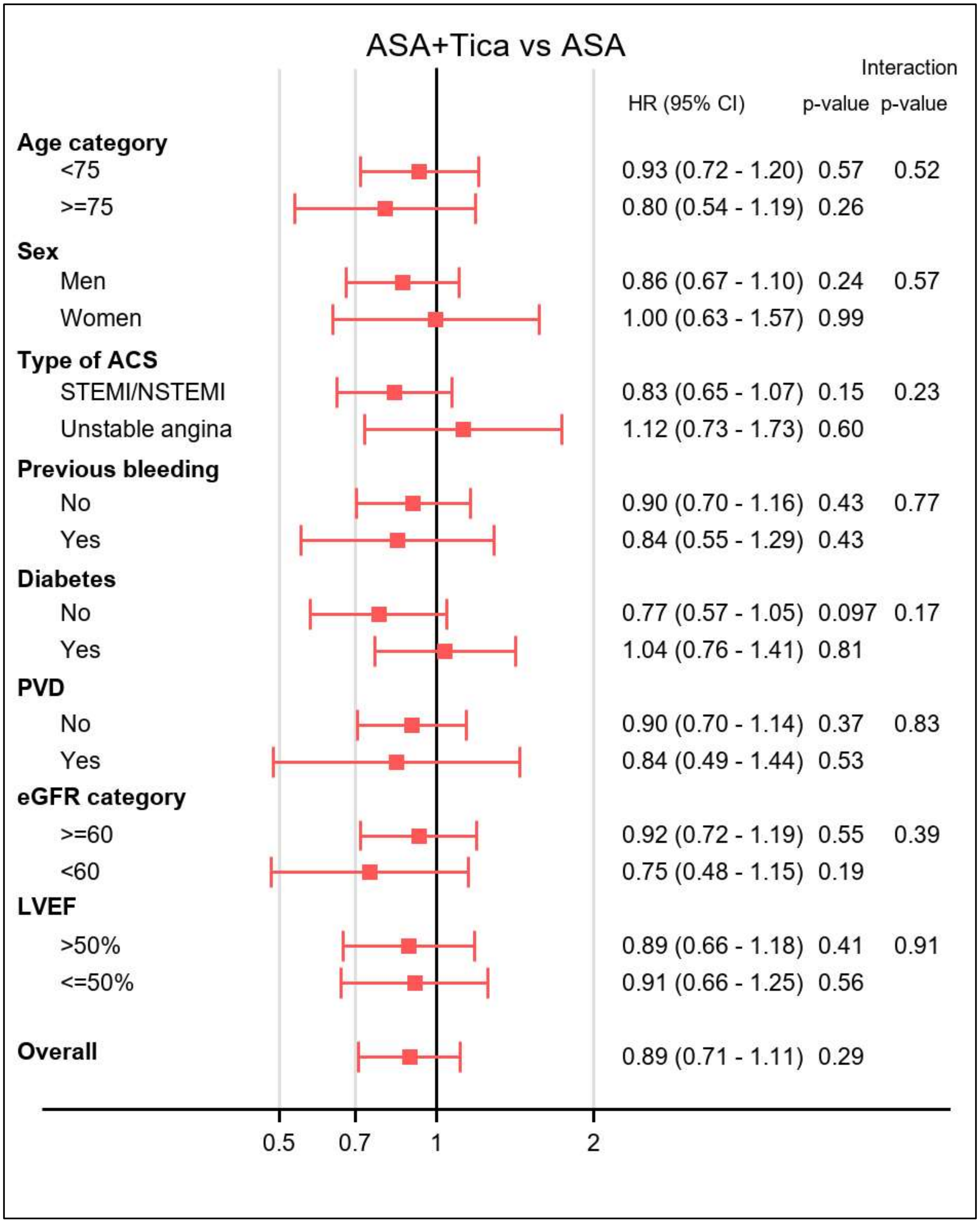

ACS, acute coronary syndrome; ASA, acetylsalicylic acid; CI, confidence interval; eGFR, estimated glomerular filtration rate; HR, hazard ratio, LVEF, left ventricular ejection fraction; NSTEMI, Non ST-elevation myocardial infarction PVD, peripheral vascular disease; STEMI, ST-elevation myocardial infarction; Tica, ticagrelor

© 2021 Björklund E et al. *JAMA Network Open.*

eFigure 5. Forest Plot Describing Multivariable Adjusted Interaction Analyses for Major Bleeding During Total Follow-up in Predefined Subgroups

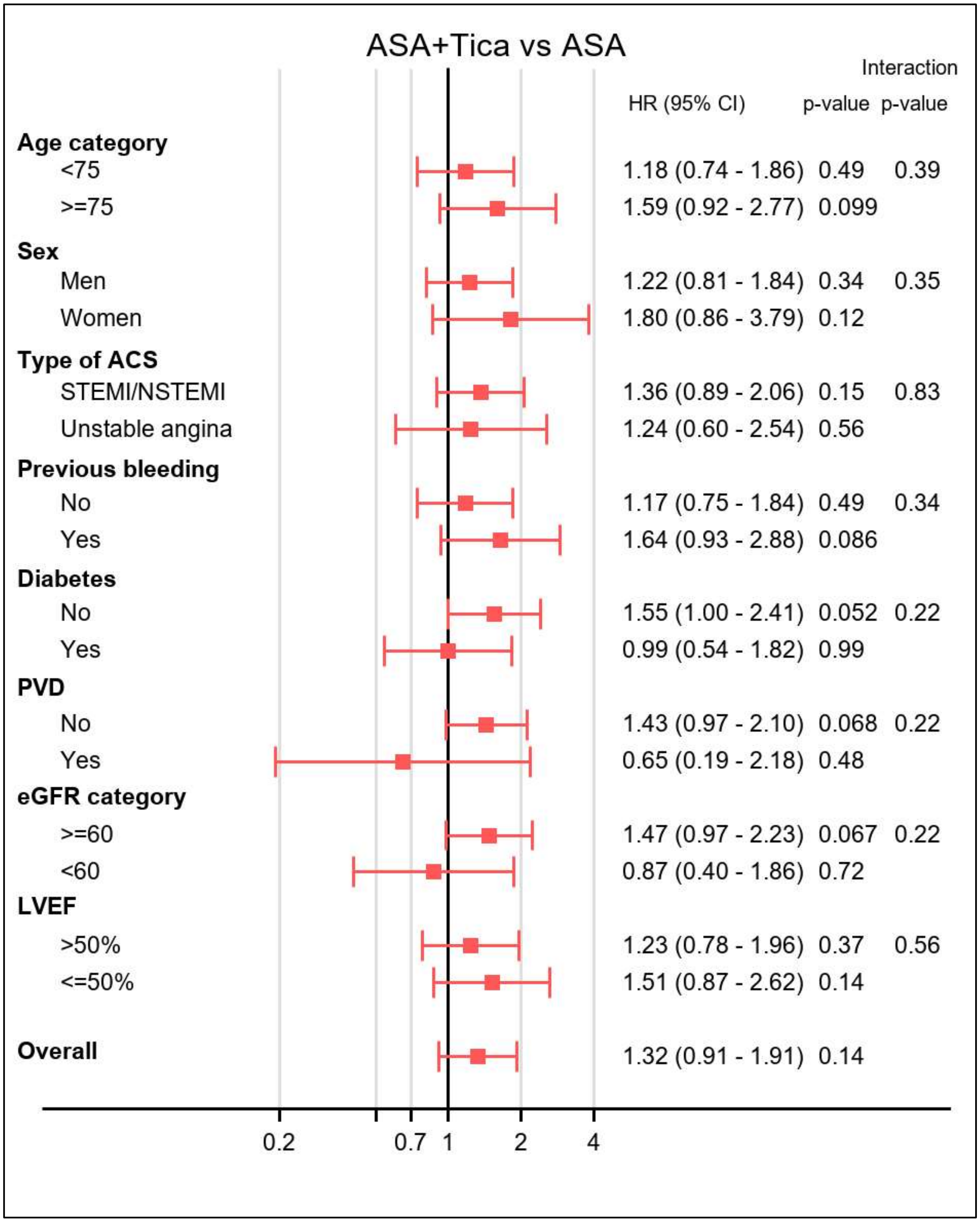

ACS, acute coronary syndrome; ASA, acetylsalicylic acid; CI, confidence interval; eGFR, estimated glomerular filtration rate; HR, hazard ratio, LVEF, left ventricular ejection fraction; NSTEMI, Non ST-elevation myocardial infarction PVD, peripheral vascular disease; STEMI, ST-elevation myocardial infarction; Tica, ticagrelor

© 2021 Björklund E et al. *JAMA Network Open.*
